# Fibroblast-Induced Paradoxical PI3K Pathway Activation in PTEN-Competent Colorectal Cancer: Implications for Therapeutic PI3K/mTOR Inhibition

**DOI:** 10.3389/fonc.2022.862806

**Published:** 2022-06-03

**Authors:** Fabiana Conciatori, Erica Salvati, Ludovica Ciuffreda, Senji Shirasawa, Italia Falcone, Francesco Cognetti, Gianluigi Ferretti, Massimo Zeuli, Donatella Del Bufalo, Chiara Bazzichetto, Michele Milella

**Affiliations:** ^1^ Medical Oncology 1, Regina Elena National Cancer Institute (IRCCS), Rome, Italy; ^2^ Preclinical Models and New Therapeutic Agents Unit, Regina Elena National Cancer Institute (IRCCS), Rome, Italy; ^3^ Institute of Molecular Biology and Pathology -National Research Council (BPM-CNR), Rome, Italy; ^4^ Department of Research, Advanced Diagnostics, and Technological Innovation (SAFU), Regina Elena National Cancer Institute (IRCCS), Rome, Italy; ^5^ Central Research Institute for Advanced Molecular Medicine, Fukuoka University, Fukuoka, Japan; ^6^ Section of Oncology, Department of Medicine, University of Verona School of Medicine and Verona University Hospital Trust, Verona, Italy

**Keywords:** PTEN, PI3K signaling, CRC, soluble factors, fibroblasts

## Abstract

**Purpose:**

Tumor-microenvironment interactions are important determinants of drug resistance in colorectal cancer (CRC). We, therefore, set out to understand how interactions between genetically characterized CRC cells and stromal fibroblasts might influence response to molecularly targeted inhibitors.

**Techniques:**

Sensitivity to PI3K/AKT/mTOR pathway inhibitors of CRC cell lines, with known genetic background, was investigated under different culture conditions [serum-free medium, fibroblasts’ conditioned medium (CM), direct co-culture]. Molecular pathway activation was monitored using Western Blot analysis. Immunoprecipitation was used to detect specific mTOR complex activation. Immunofluorescence was used to analyze cellular PTEN distribution, while different mutant PTEN plasmids were used to map the observed function to specific PTEN protein domains.

**Results:**

Exposure to fibroblast-CM resulted in increased growth-inhibitory response to double PI3K/mTOR inhibitors in PTEN-competent CRC cell lines harboring *KRAS* and *PI3K* mutations. Such functional effect was attributable to fibroblast-CM induced paradoxical PI3K/mTORC1 pathway activation, occurring in the presence of a functional PTEN protein. At a molecular level, fibroblast-CM induced C-tail phosphorylation and cytoplasmic redistribution of the PTEN protein, thereby impairing its lipid phosphatase function and favored the formation of active, RAPTOR-containing, mTORC1 complexes. However, PTEN’s lipid phosphatase function appeared to be dispensable, while complex protein-protein interactions, also involving PTEN/mTOR co-localization and subcellular distribution, were crucial for both mTORC1 activation and sensitivity to double PI3K/mTOR inhibitors.

**Data Interpretation:**

Microenvironmental cues, in particular soluble factors produced by stromal fibroblasts, profoundly influence PI3K pathway signaling and functional response to specific inhibitors in CRC cells, depending on their mutational background and PTEN status.

## Introduction

Colorectal cancer (CRC) represents the third most common malignancy worldwide (1). Despite progress in the molecular subtyping of CRC and the implementation of molecularly targeted and immunotherapy agents, metastatic disease remains largely incurable. While the epidermal growth factor (EGF) and vascular endothelial growth factor (VEGF) pathways have been successfully targeted for therapeutic purposes in advanced CRC, the prognostic and therapeutic role of other crucial signaling cascades frequently altered in CRC, such as the phosphatidylinositol 3-kinase (PI3K) pathway, remain elusive (2).

PI3K signaling regulates many oncogenic capabilities, such as protein translation, cytoskeleton remodeling, cell proliferation, and angiogenesis. PI3K phosphorylates PtdIns(4,5)P2 (PIP2) into PtdIns(3,4,5)P3 (PIP3), which acts as the second messenger for the AKT and PDK1 kinases; organization of different mTOR-containing complexes (mTORC1 and 2) signals to further effectors, such as p70^S6K1^ and 4E-binding protein (4E-BP1) downstream of the mTORC1 complex, to enable activation of the translational and transcriptional machinery. The tumor suppressor phosphatase and tensin homolog (PTEN) is the major factor restraining PI3K pathway activity and counteracting its pro-tumoral function. Collectively, aberrations of the PI3K pathway, including loss of function at the PTEN locus and its epigenetic silencing, occur in a substantial proportion of CRC cases (2). Accordingly, therapeutic inhibition of PI3K and mTOR has shown antitumor activity in preclinical models (3); however, available clinical trials with dual PI3K/mTOR inhibitors (such as PF-05212384, hereafter referred to as gedatolisib - Geda) do not recapitulate these findings (4, 5), suggesting the existence of inter- or intra-pathway feedback loops mediating therapeutic resistance. Moreover, the local inflammatory cytokine milieu and other microenvironmental factors (such as composition of the gut microbiome) may crucially interact with the genetic background of CRC and modulate its sensitivity/resistance to pathway inhibition, albeit with yet poorly defined molecular mechanisms (2).

In this context, the role of PTEN as a potential prognostic and/or predictive biomarker in CRC remains debated, not only in terms of response to established therapeutic approaches, such EGF receptor (EGFR) and/or VEGF receptor blockade (6, 7), but also in reference to experimental PI3K pathway inhibition. Indeed, a recent phase I/II study combining the mTORC1 inhibitor everolimus (Eve) with a standard mFOLFOX6/bevacizumab regimen showed promising activity in metastatic CRC, particularly in patients whose tumors lacked PTEN expression (8).

In this study, we investigated whether the presence of stromal fibroblasts may modulate sensitivity of molecularly characterized CRC cell lines to PI3K signaling inhibitors. Quite surprisingly, we observed that exposure to fibroblast-derived conditioned medium (CM) increases signaling through the PI3K/mTORC1 pathway paradoxically in PTEN-competent CRC cells, thereby sensitizing them to the growth inhibitory effect of the double PI3K/mTOR inhibitor, Geda. From a molecular standpoint, fibroblast-CM induces PTEN C-tail phosphorylation and its cytoplasmic redistribution, resulting in impaired lipid phosphatase activity; however, paradoxical PI3K/mTORC1 activation and sensitization to Geda appear to be mediated by PTEN’s protein-protein interaction, rather than lipid phosphatase activity and ability to co-localize with mTOR at the plasma membrane.

## Materials and Methods

### Cell Cultures

X-MAN™ HCT116 Parental (RRID : CVCL_HD91) and HCT116 PTEN^-/-^ (RRID : CVCL_HD92) cells were generated by Horizon from homozygous knock-out of PTEN by deleting exon 5 which encodes the active site of the protein in the CRC cell line HCT116 (Horizon Discovery www.horizondiscovery.com) (9). Isogenic cell lines HCT116, HK2–6, and HKE-3 were performed by Shirasawa’s group by *KRAS* specific targeting technique (10, 11). LS180, HT29, RKO, and SW480 cell lines were kindly provided by Dr. Federica Di Nicolantonio as previously described (11). Human foreskin fibroblasts (HFF), green fluorescent protein (GFP)-labeled fibroblasts, and BJ cell lines were kindly provided by Dr. Maurizio Fanciulli. CRC cell lines were routinely maintained in RPMI 1640 medium (Euroclone, Milan, Italy) supplemented with 10% inactivated fetal bovine serum (FBS) (Gibco, Life Technologies, California, USA), whereas HFF and BJ were routinely maintained in DMEM medium supplemented with 15% inactivated FBS. All the growth media were added with 2 mM L-glutamine (Gibco, Life Technologies, California, USA) and antibiotics (Penicinillin/Streptomycin) (Gibco, Life Technologies, California, USA); cells were grown in a humidified atmosphere with 5% CO2 at 37°C.

Normal fibroblasts (NF) were isolated from normal colon tissue, derived from patients surgically treated at Regina Elena Cancer Institute. The study was reviewed and approved by the ethics committee of the Regina Elena National Cancer Institute. Normal colon tissue was digested by 0.35% Collagenase type I (Gibco, Life Technologies, California, USA); DMEM medium supplemented with 15% inactivated FBS was added with 2X Antibiotic Antimycotic Solution (Sigma Aldrich, St. Louis, USA). Isolated fibroblasts were routinely maintained in DMEM medium supplemented with 15% inactivated FBS, added with 2 mM L-glutamine (Gibco, Life Technologies, California, USA) and antibiotics (Penicinillin/Streptomycin) (Gibco, Life Technologies, California, USA); cells were grown in a humidified atmosphere with 5% CO2 at 37°C.

Based on previous experience of our group (11), HFF-CM was obtained under standardized culture conditions: 1x10^6^ cells were seeded and after 24 h, cells were washed with PBS 1X and medium was replaced by serum-free medium (wo FBS); CM was collected after 72 h. Serum-free conditions were chosen based on the notion that production of soluble factors is tightly dependent on the type of growth media used; moreover, serum-free conditions are also necessary to characterize the pattern of expression of soluble factors released by fibroblasts (see below, paragraph 2.10), using specific ELISA and membrane-based arrays, since FBS contains basal levels of cyto/chemokines that could interfere with the detection of mediator(s) involved in the observed CM effects (12, 13).

For recombinant protein experiments, 1000 pg/mL of interleukin (IL)-8, IL-6 and monocyte chemoattractant protein (MCP)-1 were added in DMEM wo FBS. Recombinant human IL-8, IL-6, and MCP-1 were purchased from R&D Systems (R&DSystems, MN, USA).

For cell counting, Thoma chamber was used. All cell lines tested negative for mycoplasma contamination.

### Drug Treatments and Cell Proliferation Assay

Trametinib (GSK1120212, Tram) was kindly provided by GlaxoSmithKline (Brentford, Middlesex, UK). Alpelisib (BYL719, Alp), dactolisib (BEZ235, Dact), and Geda (PF05212384) were purchased from Selleck Chemicals (Huston, TX, USA). MK-2206 (MK) was kindly provided by Merck and Co. (Kenilworth, NJ, USA). Alp, Geda, MK, and Tram were dissolved in DMSO as a 1 mM (Geda, MK and Tram) and 5mM (Alp) stock solution, dact was dissolved in DMF as a 1mM. All the drugs were stored at -20°C (Alp, Dact, MK, and Tram) or -80°C (Geda). Eve (RAD001) was kindly provided from Novartis Pharma (Basel, Switzerland) and was dissolved in 100% ethanol as a 10 mM stock solution and stored at -20°C.

The final concentration of drugs was obtained by dilution with culture medium.

Effects on cell growth after 72 h of different treatments were monitored by Crystal Violet assay, as previously described (10).

### Direct Co-Cultures

X-MAN™ HCT116 Parental and HCT116 PTEN^-/-^ cells and HFF cells were seeded alone or with a 1:1 ratio in 35 mm plates (Falcon BD, Oxford, UK) in RPMI 1640 (Euroclone, Milan, Italy) supplemented with 10% inactivated FBS (Gibco, Life Technologies, California, USA), as previously described. After 24 h, culture media were replaced with serum-free DMEM (Euroclone, Milan, Italy) containing different drug concentrations alone or in combination. After 72 h of treatment, cells were suspended in ice-cold RPMI 1640 (Euroclone, Milan, Italy) supplemented with 10% inactivated FBS (Gibco, Life Technologies, California, USA). The total amount of cells was counted using the Thoma chamber and the percentage of CRC and HFF cells were analyzed by flow cytometry analysis that allows to differentiate between HFF cellular populations, fluorescently labeled in comparison to unlabeled X-MAN™ isogenic HCT116 cells.

### Plasmid and Transfection Experiments

X-MAN™ HCT116 Parental cell line was transfected with either GFP-PTEN C124S or GFP-Q399STOP plasmids, which encode for mutant PTEN C124S and PTEN Q399STOP proteins, respectively (14). Stable transfection of plasmids was performed using Lipofectamine 2000 (Invitrogen, Carlsbad, CA, USA) according to the manufacturer’s instructions.

### RNA Analysis

Total RNA was prepared from cells using the RNA extraction kit, RNeasy Mini Kit (Qiagen, Hilden, Germany) as per the manufacturer’s instructions. Of total RNA, 1 μg was converted into single-strand cDNA using Superscript II (Invitrogen, Carlsbad, CA, USA) as per the manufacturer’s instructions. Quantitative real-time PCR (RT-qPCR) was performed with Fast SYBR^®^Green quantitative PCR kit (Applied Biosystems, Foster City, CA, USA) for RPL19 (Forward primer sequence: 5′-CGGAAGGGCAGGCACAT-3′ and Reverse primer sequence 5′-GGCGCAAAATCCTCATTCTC-3′) and PIK3CD (Forward primer sequence: 5′-CCCACATGAAGAGGAACTGAGAT-3′ and Reverse primer sequence 3′- GGTTGGCAGGCTCAGTGACT-5′). Expression of PIK3CD was then normalized with RPL19.

### Western Blot Analysis

Whole cell extracts were obtained by SDS lysis buffer, containing 20 mM TrisHCl (pH 7.4) and 2% SDS, with 1X phosphatase and protease inhibitors (Thermo Fisher Scientific, Rochester, NY, USA). Protein quantification, separation, and detection were assessed as previously described (11). Membranes were probed with the primary and secondary antibodies reported in **Supplementary Materials**.

### Immunoprecipitation

Chip-Grade Protein A/G Magnetic Beads (Thermo Fisher Scientific, Rochester, NY, USA) were incubated with 2 μg of FRAP (Santa Cruz Biotechnology, Santa Cruz, CA) overnight at 4°C. Precleared beads were then incubated overnight at 4°C with 1 μg of protein. The immunoprecipitates were collected and after two washes in CHAPS buffer (containing 5 mM EDTA, 0.3% CHAPS, 50 mM Tris HCl (pH 7.4), 150 mM NaCl, 10 mM MgCl_2_, 1mM KCl, and 1X phosphatase and protease inhibitors (ThermoFisher Scientific, Rochester, NY, USA)) re-suspended in 35 µl of the same buffer and 1X Ladder buffer. The immune complexes and 20 μg of protein total cell extract were analyzed by Western blot analysis as described above.

### Immunofluorescence

5x10^4^ cells were seeded on 22x22 mm coverslips pretreated with 2% type A from porcine skin gelatin (Sigma Aldrich, St. Louis, USA); after 24 h, culture media were replaced by wo FBS DMEM (Euroclone, Milan, Italy) or HFF CM containing the indicated concentration of drug. After 24 h from treatments, cells were fixed in 4% formaldehyde in 1X PBS for 10 min, permeabilized in 1X PBS containing 0.5% Triton X-100 for 5 min at room temperature. Coverslips were incubated with primary and secondary antibodies as previously described (11). The used antibodies are reported in **Supplementary Materials**. Images were taken using Leica DMi8 microscopy (Leica, Solms, Germany) and Zeiss LSM 880 Confocal Microscope (Carl Zeiss, Oberkochen, Germany) equipped with ×63 lenses and operated by Zen 09 black software (Carl Zeiss).

### Subcellular Protein Extraction

5x10^6^ HCT116 Parental cells were seeded into 100 mm dishes (Greiner bio-one, Kremsmünster, Austria) in RPMI 1640 (Euroclone, Milan, Italy) and after 24 h from plating cell culture media were replaced by wo FBS DMEM (Euroclone, Milan, Italy) or HFF CM containing the indicated concentration of drug. After 24 h from treatment, cells were processed according to the manufacturer’s instructions of the Subcellular Protein Fractionation Kit (Thermo Fisher Scientific, Rochester, NY, USA). Membrane and cytoplasmic extract were analyzed by using Western blot assay.

### Detection and Analysis of Soluble Factor Production

3x10^5^ HFF, BJ, and NF cells were plated into 60 mm dishes (Falcon BD, Oxford, UK). After 24 h from the plating, the culture medium was replaced by wo FBS DMEM, and after 48 h from the plating media were collected and cells were counted. Cells culture media were analyzed by Human Angiogenesis Antibody Array (R&DSystems, MN, USA) according to the manufacturer’s protocol. Cell culture media were also analyzed by IL-8 and IL-6 (Enzo Life Sciences, Farmingdale, NY, USA), and MCP-1 (R&DSystems, MN, USA) specific ELISA assay, according to the manufacturer’s protocol. Absorbance was read at 450 nm. IL-8, IL-6, and MCP1 expression were represented as pg/mL and then related to the control assuming as to 100%.

### PTEN Activity ELISA Assay

6x10^6^ HCT116 Parental cells were seeded into 100 mm dishes (Greiner bio-one, Kremsmünster, Austria) in RPMI 1640 (Euroclone, Milan, Italy) and after 24 h from plating cell culture media were replaced by wo FBS DMEM (Euroclone, Milan, Italy) or HFF CM containing the indicated concentration of drug. After 24 h from treatment, cells were processed according to the manufacturer’s instructions of the PTEN activity ELISA assay (Echelon Biosciences Inc., Salt Lake City, UT, USA). Absorbance was read at 450 nm. PTEN phosphatase activity was represented as PIP2 concentration (pmol/μL) and then related to the control assuming as to 100%.

### Statistical Analysis

Results are expressed as average of three independent experiments. Results with two-tailed p values <0.05 were judged to be statistically significant. The dose of drug that causes 50% of cell growth inhibition (IC_50_) was calculated according to the Chou-Talalay method using the Calcusyn software (Biosoft, Cambridge, United Kingdom). The same software was used for deriving combination indexes (CI): the average CI at the ED50, ED75, and ED90 <1 indicates synergism, = 1 indicates additivity, and >1 indicates antagonism, respectively. Statistical analysis for co-localization was conducted using GraphPad Prism (RRID : SCR_002798) (www.graphpad.com).

## Results

### Exposure to Fibroblast-CM Sensitizes PTEN-Competent CRC Cells to Double PI3K/mTOR Inhibition

We evaluated the role of PTEN status and microenvironmental interactions in the modulation of CRC response to single or combined MAPK/PI3K inhibition. The X-MAN™ isogenic HCT116 cell lines [HCT116 Parental (PTEN-competent) and HCT116 PTEN^-/-^ (PTEN-loss)] were treated with increasing concentrations of the MEK inhibitor Tram, the PI3K/mTOR double inhibitor Geda, or their combination (Tram+Geda, 1:1 fixed dose-ratio) for 72 h under different culture conditions: serum-free medium (wo FBS), fibroblast-CM (CM HFF), or direct CRC/fibroblast co-culture (co-co), using GFP-tagged immortalized skin fibroblast (HFF). Pathway inhibitors (alone or in combination) displayed little, if any, growth inhibitory activity on isolated HFF fibroblasts (data not shown).

**Figure 1:**
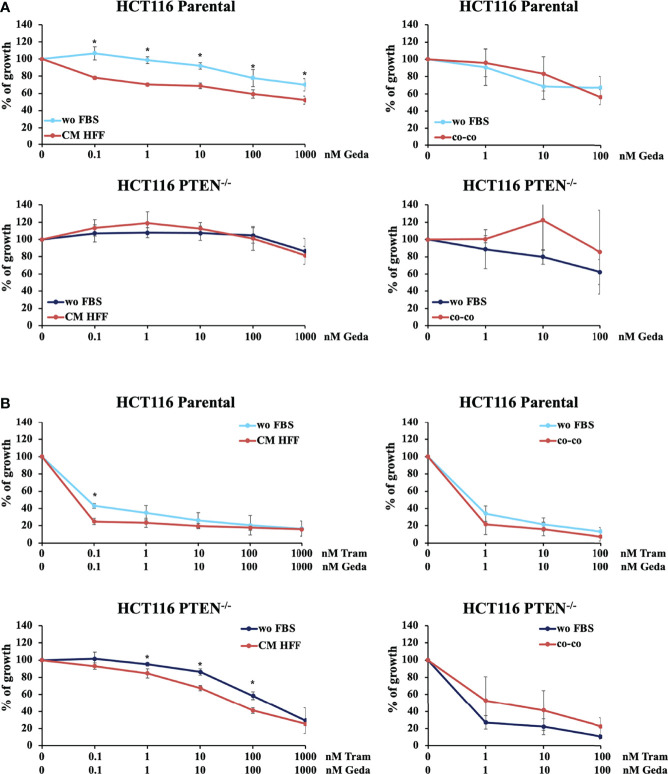
PTEN expression and microenvironmental interactions affect the response to molecular targeted inhibition in isogenic CRC cell lines. HCT116 Parental and HCT116 PTEN^-/-^ cell lines were treated with increased doses (dose range 0.1-1000 nM) of Geda or Geda and Tram combination (1:1) (**A**, **B**, respectively) in serum-free condition, fibroblast HFF derived CM (left panels) or direct CRC/GFP-tagged HFF co-culture (right panels). Cell viability for wo FBS and HFF-CM was assessed by Crystal Violet assay after 72 h of treatment. For HFF co-culture cells were counted after 72 h of treatment and the number of GFP positive and negative cells were calculated using cytofluorimetric analysis. Results are expressed as percentage of growth inhibition relative to untreated control and represent the average ± SEM of three independent experiments. Asterisks indicate statistically significant differences (p-value < 0.05 by 2-tailed Student’s t test) for the comparison between treatment under wo FBS and HFF-CM or co-co conditions.

In CRC cells, sensitivity to the growth-inhibitory activity of Tram significantly decreased in the absence of a functional PTEN (HCT116 PTEN^-/-^ cells), regardless of cell culture conditions (**Supplementary Figure S1** and **Table 1**). Conversely, exposure to fibroblast-CM specifically sensitized PTEN-competent (HCT116 Parental), but not PTEN-loss (HCT116 PTEN^-/-^) CRC cells to Geda [48% reduction in the IC_50_ in HCT116 Parental cells under CM HFF, as compared with wo FBS, conditions (**Table 1**), p-value <0.05 at all the tested doses (**Figure 1A** left panels)]; direct co-co had no effect on sensitivity to Geda, regardless of PTEN status (**Figure 1A** right panels and **Table 1**). As we previously reported (9), pharmacological interactions between Tram and Geda were highly synergistic in PTEN-loss and antagonistic in PTEN-competent cells, under isolated cell culture (wo FBS) conditions (**Figure 1B** left panels, **Supplementary Figure S2A** and **Table 1**). Exposure to HFF CM and direct co-co turned the interaction between Tram and Geda into highly synergistic in the HCT116 Parental cell line (CI: 0.01 and 0.0007, respectively), whereas only direct cell-to-cell contact influenced pharmacological interactions in HCT116 PTEN^-/-^ cells, reverting synergism (CI: 0.99) and reducing (albeit not significantly) the growth inhibitory response to combined treatment (**Figure 1B** right panels, **Supplementary Figure S2B** and **Table 1**).

**Table 1:** Response of HCT116 Parental and HCT116 PTEN^-/-^ cells to targeted inhibitors under different conditions of growth.

	Trametinib (IC_50_ nM)	Gedatolisib (IC_50_ nM)	Combo (CI)	Trametinib (IC_50_ nM)	Gedatolisib (IC_50_ nM)	Combo (CI)
	**wo FBS**			**CM HFF**		
**HCT116 Parental**	0.02	4.2x10^3^	50	0.02	2.2x10^3^	0.01
**HCT116 PTEN^-/-^**	14x10^3^	>1x10^6^	0.006	10x10^3^	>1x10^6^	0.003
	**Trametinib (IC_50_ nM)**	**Gedatolisib (IC_50_ nM)**	**Combo (CI)**	**Trametinib (IC_50_ nM)**	**Gedatolisib (IC_50_ nM)**	**Combo (CI)**
	**wo FBS**			**co-co with HFF**		
**HCT116 Parental**	0.68	0.4x10^3^	1.2	1.03	0.08x10^3^	0.0007
**HCT116 PTEN^-/-^**	24.4	0.37x10^3^	0.07	6.35	0.32x10^3^	0.99

IC_50_ and CI for all the drugs in absence of FBS (i.e. wo FBS) are intrinsically differ each other comparing to HFF-CM and co-co with HFF due to the different experimental setting conditions.

We focused on the observed sensitization to the growth-inhibitory effects of PI3K/mTOR inhibition under exposure to fibroblast-CM. First, the same functional sensitization effects observed with HFF CM were obtained using CM from another immortalized normal foreskin fibroblast cell line (BJ) and from primary fibroblasts isolated by normal colon tissue (**Supplementary Figures S3A, B**). Moreover, another double PI3K/mTOR inhibitor (Dact) exerted functional effects similar to those observed with Geda (**Supplementary Figure S4A** and **Supplementary Table S1**). Single-step inhibition along the PI3K cascade was not sufficient to obtain such functional effects: indeed, response to specific inhibitors targeting PI3K, AKT, or mTOR individually (Alp, MK, and Eve, respectively) was not modulated by exposure to fibroblast-CM in either isogenic cell line (**Supplementary Figures S4B–D** and **Supplementary Table S1**). These results confirm that exposure to normal fibroblast-CM specifically sensitizes PTEN-competent CRC cells to double PI3K/mTOR inhibition.

### Additional Genetic Determinants of Fibroblast-CM Mediated Sensitization to PI3K/mTOR Inhibition in CRC

**Figure 2:**
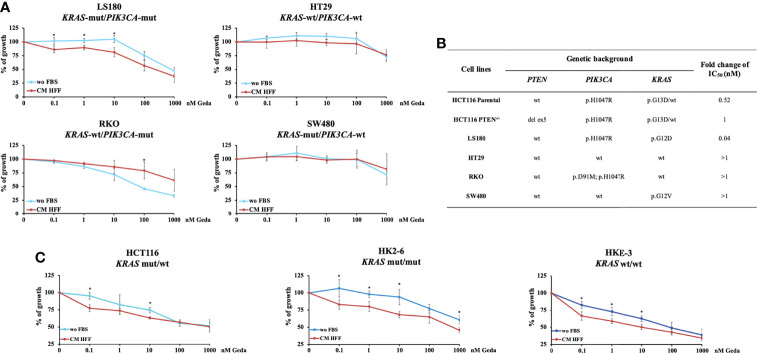
Fibroblast-CM-dependent sensitivity to Geda occurs only in *KRAS*-mut/*PIK3CA*-mut/PTEN-competent CRC cell lines. PTEN-competent LS180, HT29, RKO and SW480 cell lines and isogenic *KRAS* HCT116 cell lines (HCT116, HK2-6 and HKE-3) were treated with increasing doses of Geda (range doses 0.1-1000 nM) in serum-free condition and fibroblast HFF-CM (**A**, **C**, respectively). Results are expressed as percentage of growth inhibition relative to untreated control and represent the average ± SEM of three independent experiments. Asterisks indicate statistically significant differences (p-value < 0.05 by 2-tailed Student’s t test) for the comparison between treatment under wo FBS and HFF CM conditions. **(B)** Ratio of IC_50_ of Geda (nM) under CM HFF *vs* wo FBS condition of growth.

We next explored the effect of fibroblast-CM in modulating functional response to Geda in four PTEN-competent CRC cell lines (LS180, HT29, RKO, and SW480), characterized by different genetic background (*KRAS*-mut/*PIK3CA*-mut, *KRAS*-wt/*PIK3CA*-wt, *KRAS*-wt/*PIK3CA*-mut, and *KRAS*-mut/*PIK3CA*-wt, respectively) (**Figures 2A, B**). Each cell line was exposed to increasing concentrations of Geda (0.1-1000 nM) under wo FBS or HFF CM conditions for 72 h. Similar to HCT116 Parental cells, only LS180 (*PIK3CA*-mut/*KRAS*-mut/PTEN-competent) cells displayed a statistically significant difference in their response to Geda according to culture conditions (p-value < 0.05 for the lowest concentrations of Geda; **Figures 2A, B**). Conversely, in the *KRAS*-wt/*PIK3CA*-mut RKO cell line exposure to fibroblast-CM induced significant resistance to Geda at the highest concentrations. No significant differences were observed in the HT29 and SW480 cell lines (**Figure 2A**).

Since HCT116 cells are known to express high levels of the p110δ PI3K isoform (15), we also evaluated whether the pattern of p110δ PI3K expression correlated with functional response to Geda in the presence or absence of HFF-CM. As shown in **Supplementary Figure S4E**, p110δ PI3K (*PIK3CD*) expression by RT-qPCR was highest in HCT116 Parental and SW480 cells when compared in a panel of five cell lines, and did not correlate with HFF-CM induced functional sensitization to Geda-mediated growth inhibition.

We next investigated the response of isogenic *KRAS* HCT116 cell lines (parental HCT116 - heterozygous *KRAS*-mut, HK2-6 - homozygous *KRAS*-mut, and HKE-3 cell lines - homozygous *KRAS*-wt, respectively) to Geda under different culture conditions. As shown in **Figure 2C**, the most pronounced fibroblast-CM dependent functional sensitization to Geda was observed in the homozygous *KRAS*-mut isogenic cell line HK2-6. Overall, these results suggest that, in addition to the expression of a functional PTEN protein, the mutational status of both *KRAS* and *PIK3CA* plays a role in the observed functional sensitization to double PI3K/mTOR inhibition induced by fibroblast-CM in CRC cells.

### Fibroblast-CM Induces Paradox PI3K Pathway Activation Which Can be Reverted by Double PI3K/mTOR Inhibition in PTEN-Competent CRC Cell Lines

**Figure 3:**
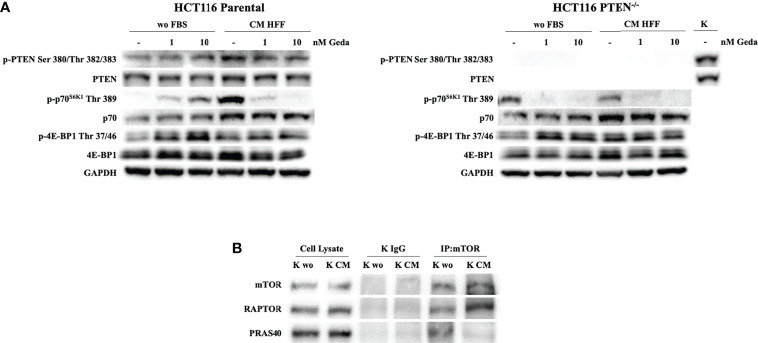
Evaluation of PI3K pathway activation in response to Geda in the presence of fibroblast-derived CM. **(A)** HCT1116 Parental and HCT116 PTEN^-/-^ cell lines were treated with 1 and 10 nM of Geda in serum-free condition and HFF-CM. Cells were lysed and analyzed by Western Blotting using specific antibodies (as indicated). GAPDH is shown as protein loading and blotting control. **(B)** HCT116 Parental cells were grown in wo FBS and HFF CM for 24 h. Endogenous mTOR was immunoprecipitated (IP:mTOR) and the immunocomplexes were blotted for RAPTOR and PRAS40. mTOR, RAPTOR and PRAS40 levels in total cell lysates are also shown. Results of a representative experiment out of three independent experiments performed are shown.

We next investigated whether fibroblast-CM might influence PTEN expression and function and PI3K pathway activation. Exposure of HCT116 Parental cells (PTEN-competent) to fibroblast-CM for 24 h induced PTEN phosphorylation at Ser 380/Thr 382/383, along with relative protein accumulation, and p70^S6K1^ phosphorylation at Thr 389, with relative protein accumulation; under these conditions, double PI3K/mTOR inhibition by Geda paradoxically induced PTEN and p70^S6K^ phosphorylation under unstimulated conditions, while substantially decreasing their phosphorylation induced by fibroblast-CM (**Figure 3A** left panel). A similar modulation in p-PTEN Ser 380/Thr 382/383 and p70^S6K1^ Thr 389 in response to both fibroblast-CM exposure and Geda treatment was also observed in the PTEN-competent LS180 cell line (**Supplementary Figure S5A**) and comparable results were observed using a different double PI3K/mTOR inhibitor (Dact) in HCT116 Parental cells (**Supplementary Figure S5B**). Conversely, in HCT116 PTEN^-/-^ cells p70^S6K1^ Thr 389 phosphorylation levels were similar under both unstimulated and fibroblast-CM stimulated conditions and were consistently decreased by Geda under either culture condition (**Figure 3A** right panel). Modulation in other pathway elements (such as reduction in p-mTOR Ser 2448, Ser 2481, and Thr 2446, and total mTOR protein; increase in p-4E-BP1 Thr 37/46 and total 4E-BP1 protein levels) upon exposure to fibroblast-CM was consistent between PTEN-competent (HCT116 Parental, left panel) and PTEN-loss (HCT116 PTEN^-/-^, right panel) isogenic CRC cells (**Supplementary Figure S5C**).

Among soluble factors potentially involved in the fibroblast-CM induced PI3K pathway stimulation in PTEN-competent CRC cells, IL-8, IL-6, and MCP-1 were the most prominently produced by all three normal fibroblast cell lines tested (HFF, BJ, and NF; **Supplementary Figure S5D**); however, exogenous addition of recombinant IL-8, IL-6, or MCP-1 (alone or in combination) was not able to induce p70^S6K1^ phosphorylation at Thr 389, despite upregulation of p-PTEN Ser 380/382/383 levels (**Supplementary Figure S5E**).

To further investigate the mechanisms underlying paradox PI3K pathway activation in response to fibroblast-CM stimulation in PTEN-competent cells, we examined mTOR complex formation under different culture conditions. Co-immunoprecipitation experiments shown in **Figure 3B** indicated specific binding of RAPTOR to, and dissociation of, PRAS40 from mTOR under fibroblast-CM stimulated conditions.

Overall, such evidence indicates that yet unidentified soluble factors released by normal fibroblasts paradoxically activate the PI3K axis in PTEN-competent cells, by specifically activating the mTORC1 complex.

### Fibroblast-CM-Induced Paradox PI3K Pathway Activation Is Mediated by PTEN Intracellular Redistribution and Inactivation

**Figure 4:**
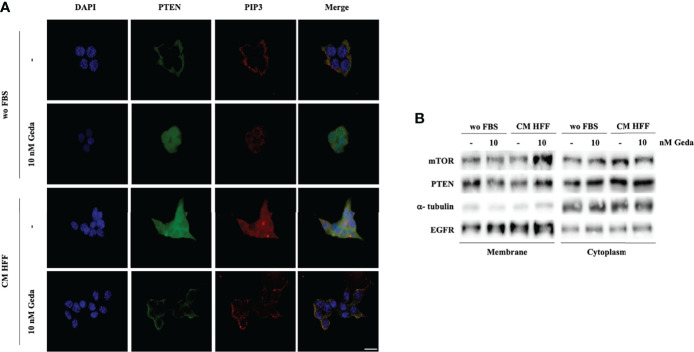
Fibroblast-derived CM induces the reduction of PTEN activity and its re-distribution in the cell. **(A)** Direct immunofluorescence analysis of cellular localization of PTEN (green) and PIP3 (red). Nuclear staining was evaluated with DAPI in HCT116 Parental cells in both wo FBS and HFF CM, in absence or presence of 10 nM Geda. Results of a representative experiment out of three independent experiments performed are shown. Microphotographs were taken at ×63 magnification. Scale bar 50 μm. **(B)** HCT116 Parental cell line were treated with 10 nM of Geda in wo FBS and HFF-CM conditions. Membrane and cytoplasmic fractions of cell line were isolated; molecular differences were analyzed by Western Blotting using specific antibodies (EGFR and α-tubulin are shown as protein loading and blotting control for membrane and cytoplasmic extract, respectively). Results of a representative experiment out of three independent experiments performed are shown.

As shown in **Figure 3A**, fibroblast-CM induced prominent phosphorylation of PTEN’s C-tail (Ser 380/Thr 382/383) in PTEN-competent CRC cells. Consistent with the well-known role of PTEN phosphorylation in modulating protein localization and activity, immunofluorescence experiments conducted in HCT116 Parental cells showed predominantly membrane-bound PTEN under basal conditions (wo FBS), while its distribution became spread and predominantly cytoplasmic upon exposure to either Geda or fibroblast-CM (**Figure 4A**); under these conditions, PIP3 distribution essentially paralleled that of PTEN protein, even though the spread, cytoplasmic distribution and relative abundance of PIP3 were less evident in Geda-treated, as compared with fibroblast-CM treated, cells. Regardless, under fibroblast-CM stimulated conditions Geda treatment restored membrane-bound PTEN/PIP3 localization (**Figure 4A**). These findings were further confirmed by cell fractionation experiments (**Figure 4B**): indeed, PTEN protein decreased in the membrane-bound fraction and increased in the cytoplasmic fraction upon exposure to either Geda or fibroblast-CM and accumulated back in the membrane fraction upon combined fibroblast-CM/Geda treatment; interestingly, the mTOR protein changed its subcellular distribution in parallel to that of PTEN (**Figure 4B**). However, when PTEN enzymatic activity was measured in whole cell lysates in the presence of exogenous PIP3 by ELISA (thus not reflecting the potential role of PTEN subcellular localization), Geda treatment did not significantly affect PTEN lipid phosphatase activity under basal (wo FBS) conditions, while exposure to fibroblast-CM reduced enzymatic activity by approximately 50%; combined fibroblast CM/Geda treatment restored PTEN activity to basal levels (**Supplementary Figure 6**).

Overall, these data suggest that fibroblast-CM induced PI3K pathway activation is due, at least in part, to PTEN C-tail phosphorylation, subcellular redistribution, and impaired enzymatic activity, all of which can be restored by simultaneous PI3K/mTOR inhibition.

### Protein-Protein Interaction PTEN Domain Mediates Fibroblast-CM-Dependent Paradox PI3K Activation and Sensitization to Geda

Taken together, the above-described results indicate that the presence of a functional PTEN protein is necessary for fibroblast-CM mediated paradox PI3K pathway activation and, hence, to sensitize CRC cells to the growth-inhibitory effects of double PI3K/mTOR inhibition. To dissect whether these molecular and functional effects are mediated by PTEN protein-protein interaction or lipid phosphatase domains, HCT116 Parental cells were stably transfected with two chimeric GFP-mutant PTEN genes exerting a dominant negative function: the Q399STOP construct produces a PTEN protein lacking the PDZ-domain and protein-protein interaction ability; the C124S construct produces a PTEN protein lacking enzymatic activity and the ability to dephosphorylate both proteins and lipids (14, 16, 17).

**Figure 5:**
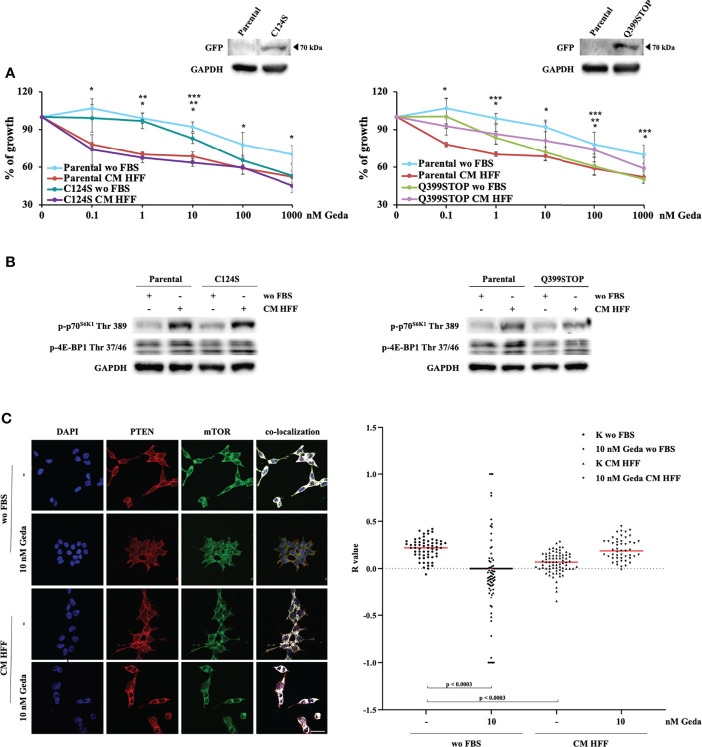
PDZ PTEN domain mediates the effect of fibroblast-CM. **(A)** HCT116 Parental clones stably transfected with either a plasmid encoding PTEN C124S (C124S, left panel) or PTEN Q399STOP (Q399STOP, right panel) were treated with increasing doses of Geda (0.1-100 nM) in wo FBS or HFF-CM conditions. Cell viability was assessed by Crystal Violet assay after 72 h of treatment. Results are expressed as percentage of growth inhibition relative to untreated control and represent the average ± SEM of three independent experiments. Asterisks indicate statistically significant differences (p-value < 0.05 by 2-tailed Student’s t test) for the comparison between different conditions (*, **, *** for Parental wo FBS *vs* HFF-CM conditions, Clones (i.e. C124S or Q399STOP) wo FBS *vs* HFF-CM, Parental wo FBS *vs* Clones wo FBS conditions, respectively). **(B)** HCT1116 Parental, C124S clone (left panel) and Q399STOP clone (right panel) cell lines were grown under wo FBS and HFF-CM conditions. Cells were lysed and analyzed by Western Blotting using specific antibodies (as indicated). GAPDH is shown as protein loading and blotting control. Results of a representative experiment out of three independent experiments performed are shown. **(C)** Colocalization analysis (representative images with white spots in left panel) confirmed a clear association in wo FBS condition and the lack of a specific association in HFF-CM and 10 nM Geda wo FBS condition. Microphotographs were taken at ×63 magnification. Scale bar: 50 μm. For the total sample (right panel), R values for control and 10 nM Geda in each condition of growth (wo FBS and HFF-CM) were analyzed by parametric unpaired *t*-test with Welch’s correction. PTEN/mTOR codistribution showed a significant pattern of superimposition in wo FBS condition as compared to both HFF-CM control condition and 10 nM Geda wo FBS condition. Results of a representative experiment out of three independent experiments performed are shown.

C124S transfection did not affect response to Geda under either basal (wo FBS) or fibroblast-CM stimulated conditions (**Figure 5A**, left panel). Conversely, Q399STOP transfection completely abrogated the Geda-sensitizing effect of fibroblast-CM: indeed, response to Geda was not significantly different between fibroblast-CM stimulated and unstimulated Q399STOP-transfected cells; however, Q399STOP-transfected cells were significantly more sensitive to Geda than their Parental counterpart under unstimulated conditions and less sensitive under fibroblast-CM stimulated conditions (**Figure 5A**, right panel). Molecular endpoints were modulated consistently with the observed functional effects: HFF CM-dependent stimulation of p70^S6K1^ Thr 389 and 4E-BP1 Thr 37/46 phosphorylation was less pronounced in Q399STOP-transfected cells, as compared to HCT116 Parental and C124S-transfected cells (**Figure 5B**), suggesting a more prominent role for PTEN protein-protein interaction, rather than phosphatase, activity in fibroblast-CM induced paradox PI3K pathway activation. Together with evidence of fibroblast-CM mediated mTORC1 formation only in PTEN-competent cells and concomitant redistribution of both PTEN and mTOR upon treatment, this observation led us to investigate whether PTEN could physically interact with mTOR. To this purpose, we evaluated PTEN and mTOR co-localization under different culture conditions using confocal microscopy. As shown in **Figure 5C**, a substantial pattern of superimposition between the two proteins was observed in unstimulated HCT116 Parental cells (wo FBS); exposure of HCT116 Parental cells to either Geda or fibroblast-CM significantly reduced the pattern of superimposition (p<0.0003 for both), which returned to basal when fibroblast-CM stimulated cells were exposed to Geda (p-value for the comparison between HCT116 Parental cells wo FBS and HCT116 Parental cells HFF-CM+Geda not significant), closely paralleling data on subcellular PTEN localization.

## Discussion

In this study we provide evidence that PTEN distribution and function influence the response to targeted therapies according to the presence of stromal elements. Exposure to fibroblast-CM paradoxically hyperactivates the PI3K signaling cascade in *PIK3CA-*mut/*KRAS*-mut/PTEN-competent CRC cells, resulting in increased sensitivity to the growth inhibitory effects of pharmacologic double PI3K/mTOR blockade. Such effect appears to be crucially mediated by PTEN phosphorylation and subcellular redistribution and increased mTORC1 formation and signaling, which, in turn, appear to be related to PTEN’s protein-protein interaction (rather than enzymatic) activity and, possibly, physical interaction with mTOR.

Available evidence indicates a predominant role of cancer cell’s genetic background in dictating the response to MAPK signaling inhibitors (10, 18–22). In line with previous evidence from our group (9, 23), sensitivity to MEK inhibition was indeed dependent on functional PTEN expression, regardless of the condition of growth of CRC cells. Consistently, the combination of MAPK and PI3K inhibitors synergistically decreased cell viability only in PTEN-loss contexts (9). Nevertheless, the effects of PI3K pathway inhibition (alone or combined with MEK inhibition) on CRC cell growth are profoundly influenced by the presence of fibroblast-derived soluble factors or direct cell-to-cell contact, particularly in PTEN-competent cells. In HCT116 PTEN^-/-^ cells direct co-culture with stromal fibroblasts only slightly modified the qualitative interaction between Tram and Geda and exposure to fibroblast-CM did not affect their relative resistance to single agent Geda. Conversely, in PTEN-competent HCT116 Parental cells both direct co-culture and exposure to fibroblast-CM changed the interaction between Tram and Geda from antagonistic/additive to strongly synergistic. Most importantly, exposure to fibroblast-CM sensitized PTEN-competent CRC cells to the growth inhibitory effects of double PI3K/mTOR inhibitors.

In a quest to understand the molecular mechanisms underlying such sensitization, we found that yet unidentified soluble factors released by normal fibroblasts induce activation of the PI3K/mTOR axis in CRC cells. Such selective activation occurs only in PTEN-competent cells, a rather paradoxical finding, since the main known function of PTEN is to negatively regulate PI3K activity, through modulation of PIP3 availability (24, 25). Indeed, PTEN inactivation (by genetic, epigenetic, or post-translational events) is usually required to elicit the activation of PI3K and its downstream effectors, such as AKT, mTOR, and p70^S6K1^ (26). mTOR, in particular, can participate in two different enzyme complexes, mTORC1 and mTORC2, with distinct signaling and functional activities (27). In our experimental model, fibroblast-CM specifically elicits mTORC1 activation, shifting the balance toward mTOR association with the RAPTOR scaffold protein and displacing the inhibitor protein PRAS40 from the complex (28, 29). The imbalance toward mTORC1 signaling induced by exposure to fibroblast-CM is supported by unequivocal phosphorylation of p70^S6K1^ (and to a lesser extent 4E-BP1), which is considered to be the main effector downstream of the mTORC1 (30, 31). On the other hand, fluctuations in p-AKT Ser 473 phosphorylation levels in cell lysates do not necessarily reflect, nor should be considered an accurate reading of, the activation status of mTOR (32) and were therefore not considered in this context.

Perhaps the most unexpected finding is that fibroblast-CM mediated PI3K/mTORC1 activation paradoxically requires an intact PTEN protein, in that it is not observed in PTEN^-/-^ CRC cells. In our HCT116 Parental cells model, exposure to fibroblast-CM inactivates PTEN through its C-tail phosphorylation and consequent cellular redistribution, thereby impairing its lipid phosphatase activity (33–35). However, PTEN’s phosphatase domain, which is lost in the C124S mutants (14, 36, 37), appears to be dispensable for fibroblast-CM induced p70^S6K1^ Thr 389 phosphorylation and functional sensitization to Geda. On the contrary, loss of the protein-protein interaction PDZ domain [lost in the Q399STOP mutants; (14)] reduces the ability of fibroblast-CM to activate the PI3K/mTORC1 signaling axis and completely abrogates functional sensitization to Geda’s growth inhibitory effects. The observed C-terminal PTEN phosphorylation caused by fibroblast-CM stimulation affects amino acid residues (Ser 380/Thr 382/383) within the PDZ binding domain (33). Thus, in addition to subcellular redistribution and loss of lipid phosphatase activity, this may result in changes in the composition of PTEN-associated macromolecular complexes (PAC). It has indeed been shown that PI3K p85/p110β selectively associates with unphosphorylated PTEN within the PAC, raising the possibility that PTEN restrains tumor-promoting PI3K activity not only through its established lipid phosphatase activity, but also through specific protein-protein interactions (38). Co-localization experiments shown in **Figure 5** suggest mTOR itself could be part of phosphorylated PTEN-orchestrated PAC; however, the details of such putative interaction remain to be elucidated, since we could not provide direct evidence of physical interaction between PTEN and mTOR and interactomic databases do not describe such interaction (www.ebi.ac.uk/intact/interactions; www.string-db.org/cgi/input).

**Figure 6:**
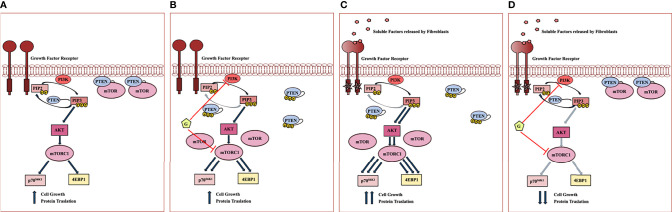
Working model of fibroblast mediated PI3K pathway hyperactivation and response to double PI3K/mTOR inhibitors in PTEN-competent CRC cell lines. **(A)** In serum-free condition of growth, PTEN is associated to plasma membrane and co-localizes with mTOR, acting as a negative regulator of PI3K signaling at different levels. **(B)** Despite PI3K inhibition, Geda (G) causes PTEN C-terminal tail phosphorylation and cytoplasmic redistribution, accompanied by loss of co-localization with mTOR at the plasma membrane, presumably by interrupting a negative feedback loop; the net result of double PI3K/mTOR inhibition, in this context, is paradoxical pathway (re)activation, whereby Geda induces its own resistance mechanism(s). **(C)** Upon fibroblast-CM exposure, two main molecular events concurrently occur with the PTEN C-terminal tail phosphorylation and redistribution within the cells: I) PTEN loses its negative control on PIP3 phosphorylation; II) PTEN no longer co-localizes with mTOR at the plasma membrane, thus rendering mTOR more available to form functionally active complexes and causing mTORC1 and downstream pathway hyperactivation. **(D)** Under conditions in which fibroblast-CM drives PTEN-dependent PI3K/mTOR paradoxical activation, double PI3K/mTOR inhibition strongly switches off signaling through the PI3K cascade, thereby restoring Geda-mediated growth inhibition.

Interrelationships between PI3K, PTEN, AKT and mTOR subcellular localization and enzymatic activity are extremely complex. The data presented on the effects of Geda and fibroblast-CM, alone or combined, in HCT116 Parental cells expressing a functional PTEN protein support the following model (**Figure 6**) when used alone, both Geda and fibroblast-CM induce PTEN cytoplasmic redistribution, loss of PTEN/mTOR co-localization, and paradoxical pathway (re)activation; however, although the result is functionally similar (paradoxical pathway activation), the molecular mechanisms of the observed phenomena might be different between Geda and fibroblast-CM: indeed, under conditions in which pathway activation is induced by exposure to fibroblast-CM, Geda regains its ability to functionally inhibit the pathway and effectively counteract tumor cell growth. Overall, these findings suggest that under fibroblast-CM unstimulated conditions Geda induces its own resistance mechanism by, presumably, interrupting a negative feedback loop and paradoxically causing pathway (re)activation. This is reminiscent of the paradoxical MAPK activation observed BRAF-selective kinase inhibitors in *BRAF*-mut cancer models, whereby a kinase-inhibited BRAF protein is still able to form complexes with CRAF or yet unidentified interactors and paradoxically activate MEK and ERK downstream (10, 18, 19). Similar to that functional situation, which requires double MEK/ERK blockade to shut the pathway down and support clinical activity (20–22), here we show that fibroblast-CM induced paradoxical PI3K/mTORC1 specifically sensitizes PTEN-competent CRC cells to double PI3K/mTOR, but not to single PI3K, AKT, or mTOR, inhibition.

Overall, these results are consistent with a PTEN-centered bidirectional crosstalk between stromal and cancer cells (39). Indeed, PTEN can act as a paracrine regulator of the PI3K pathway: secreted PTEN-long, a translational variant of PTEN, can modulate PI3K activity in PTEN-loss bystander cells (40, 41). On the other hand, exosomes released by hepatocellular carcinoma cells inactivate PTEN in hepatic stellate cells, hence converting them into cancer associated fibroblasts involved in cancer progression (42). Moreover, PTEN-loss in stromal fibroblasts induces miR-320 downregulation and activates an oncogenic secretome, which in turn promotes tumor angiogenesis and tumor-cell invasion in breast cancer models (43).

The mutational status of the key genes involved in specific molecular cascades (e.g. *PIK3CA*, *KRAS*, PTEN) remains a crucial predictor of targeted drug response and it is now well established that feedback loops and pathway crosstalks are involved in conferring primary and/or acquired drug resistance, even for PI3K signaling inhibitors (44). A recent review by Brandao and coworkers critically analyzes clinical trials with PI3K inhibitors and suggests *KRAS* mutations among potential biomarkers for resistance in *PIK3CA*-mut breast cancer (45, 46). In our model, both *KRAS* and *PIK3CA* mutational status affected response to the double PI3K/mTOR inhibition in the presence of fibroblast-CM. Interestingly, mounting evidence indicates that oncogenic *KRAS* mutations in cancer cells shape tumor microenvironment composition and affect the properties and functions of its constituents (11, 47). This is of paramount importance from a clinical standpoint, since mutational status is regarded as a relatively stable characteristic in CRC and other tumors and better understanding of such relationships would provide clinicians with reliable biomarkers to select patients at the highest chance of benefit from specific targeted treatments. *KRAS* and *PIK3CA* mutations occur concomitantly in approximately 10%-12% of CRC and are significantly associated (p-value < 0.001; data retrieved from cBioportal (www.cbioportal.org); (48, 49), indicating that the findings described here might be relevant for a significant fraction of CRC patients, considered resistant to currently available treatments.

Altogether, we describe here a novel molecular circuitry, whereby soluble mediators released by microenvironmental elements (fibroblasts) modulate PTEN activity in CRC cells, causing paradoxical PI3K/mTORC1 activation and resulting in increased response to specific double PI3K/mTOR inhibitors in specific genetic contexts (*KRAS*-mut/*PIK3CA*-mut/PTEN-competent). The exact definition of the molecular mechanisms underlying such crosstalk deserves further investigation and may provide clues to the selection of CRC patients at the highest chance to benefit from PI3K pathway inhibitors and provide the rationale for novel therapeutic approaches.

## Data Availability Statement

The raw data supporting the conclusions of this article is available in “**Table 1**” in the **Supplementary Material**, further inquiries can be directed to the corresponding author/s.

## Author Contributions

FCon, CB, and MM contributed to conception and design of all the experiments, supervised data acquisition and analysis, and wrote the manuscript. FCon and CB performed experiments, contributed to data acquisition, analysis and interpretation of the results. ES, LC, SS, and IF performed experiments and contributed to data acquisition and analysis. FCog, GF, MZ, and DB provided critical reagents, contributed to conception and critically revised the manuscript. All authors reviewed and gave final approval.

## Funding

This work was supported in part by Bando Interno Ricerca Corrente IRE 2021 (Fabiana Conciatori), IFO Sperimentazioni OM1, AIRC 5 x mille Multiunit Extension (Grant #9979, Michele Milella), and Fondo Universitario per la Ricerca (FUR) University of Verona (Michele Milella). Chiara Bazzichetto was supported by an AIRC fellowship for Italy.

## Conflict of Interest

The authors declare that the research was conducted in the absence of any commercial or financial relationships that could be construed as a potential conflict of interest.

## Publisher’s Note

All claims expressed in this article are solely those of the authors and do not necessarily represent those of their affiliated organizations, or those of the publisher, the editors and the reviewers. Any product that may be evaluated in this article, or claim that may be made by its manufacturer, is not guaranteed or endorsed by the publisher.
