# Supplementary material for: Fibroblast-Induced Paradoxical PI3K Pathway Activation in PTEN-Competent Colorectal Cancer: Implications for Therapeutic PI3K/mTOR Inhibition
Source: Front Oncol. 2022 Jun 3;12:862806. doi: 10.3389/fonc.2022.862806 (PMC9203999; doi:10.3389/fonc.2022.862806)

1. Supplementary Material

1.1. Western blot analysis

Membranes were probed with the following primary antibodies:
phospho-mTOR Ser 2448 (Cell Signaling Technology Cat# 2971, RRID:AB_330970), phospho-mTOR Ser 2481 (Cell Signaling Technology Cat# 2974, RRID:AB_2262884), phospho-mTOR Thr 2446 (Millipore Cat# 09-345, RRID:AB_1163477), mTOR (Cell Signaling Technology Cat# 2972, RRID:AB_330978), RAPTOR (Cell Signaling Technology Cat# 4978, RRID:AB_2893248), PRAS40 (Cell Signaling Technology Cat#2691, RRID:AB_2225033), phospho-PTEN Ser 380/Thr 382/383 (Cell Signaling Technology Cat# 9554, RRID:AB_331411), PTEN (Cell Signaling Technology Cat# 9552, RRID:AB_10694066), phospho-p70^{S6K1} Thr 389 (Cell Signaling Technology Cat# 9205, RRID:AB_330944), p70^{S6K1} (Cell Signaling Technology Cat# 9202, RRID:AB_331676), phospho-4E-BP1 Thr 37/46 (Cell Signaling Technology Cat# 2855, RRID:AB_560835), 4E-BP1 (Cell Signaling Technology Cat# 9644, RRID:AB_2097841), GAPDH (Cell Signaling Technology Cat# 5174, RRID:AB_10622025), α-tubulin (Cell Signaling Technology Cat# 2144, RRID:AB_2210548), EGFR (Cell Signaling Technology Cat# 3197, RRID:AB_1903955), GFP (Cell Signaling Technology Cat# 2555, RRID:AB_10692764).

1.2 Immunofluorescence

Coverslips were incubated with following primary antibodies: mTOR (Millipore Cat# OP97-100UG, RRID:AB_476537), PIP3 (Echelon Biosciences Inc. Cat# Z-P345b, RRID:AB_11127183) PTEN (Cell Signaling Technology Cat# 9552, RRID:AB_10694066).
Coverslips were incubated with the following secondary antibodies: goat anti-rabbit Alexa Fluor 488 (Thermo Fisher Scientific Cat# A-11034, RRID:AB_2576217) and goat anti-mouse Alexa Fluor 594 (Thermo Fisher Scientific Cat# A-11032, RRID:AB_2534091) for images shown in Figure 4A, goat anti-mouse Alexa Fluor 488 (Cell Signaling Technology Cat# 4408, RRID:AB_10694704) for images shown in Figure 5C, goat anti-rabbit Alexa Fluor 555 (Cell Signaling Technology Cat# 4413, RRID:AB_10694110) for images shown in Figure 5C.

2. Supplementary Figures

Supplementary Figure 1

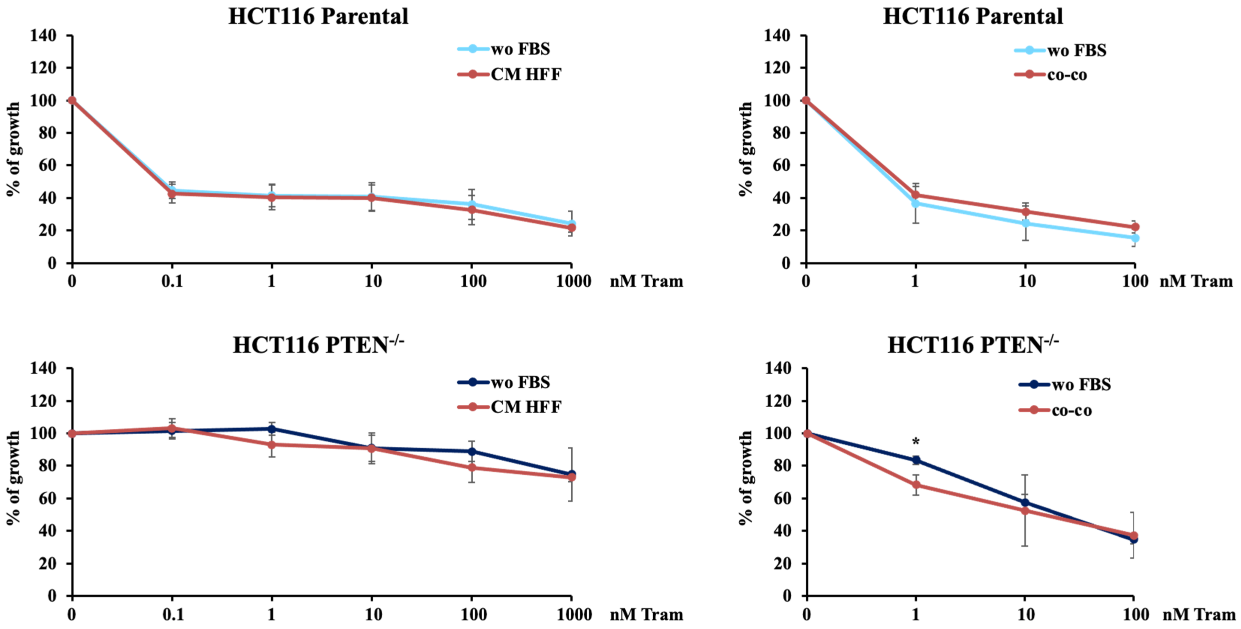

Supplementary Figure 1. PTEN expression affects the response to Tram inhibition in isogenic CRC cell lines regardless of microenvironmental interactions. The response to MEK inhibitor (Tram) was principally dictated by genetic background of isogenic CRC cells. HCT116 Parental and HCT116 PTEN$^{-/-}$ cell lines were treated with increased doses (dose range 0.1-1000 nM) of Tram in serum-free condition, fibroblast HFF derived CM (left panels) or direct CRC/GFP-tagged HFF co-culture (right panels). Cell viability for wo FBS and HFF CM were assessed by Crystal Violet assay after 72 h of treatment. For HFF co-culture cells were counted after 72 h of treatment and the number of GFP positive and negative cells were calculated using cytofluorimetric analysis.
Results are expressed as percentage of growth inhibition relative to untreated control and represent the average ± SEM of three independent experiments. Asterisks indicate statistically significant differences (p-value < 0.05 by 2-tailed Student's t test) for the comparison between treatment under wo FBS and HFF CM or co-co conditions.

Supplementary Figure 2

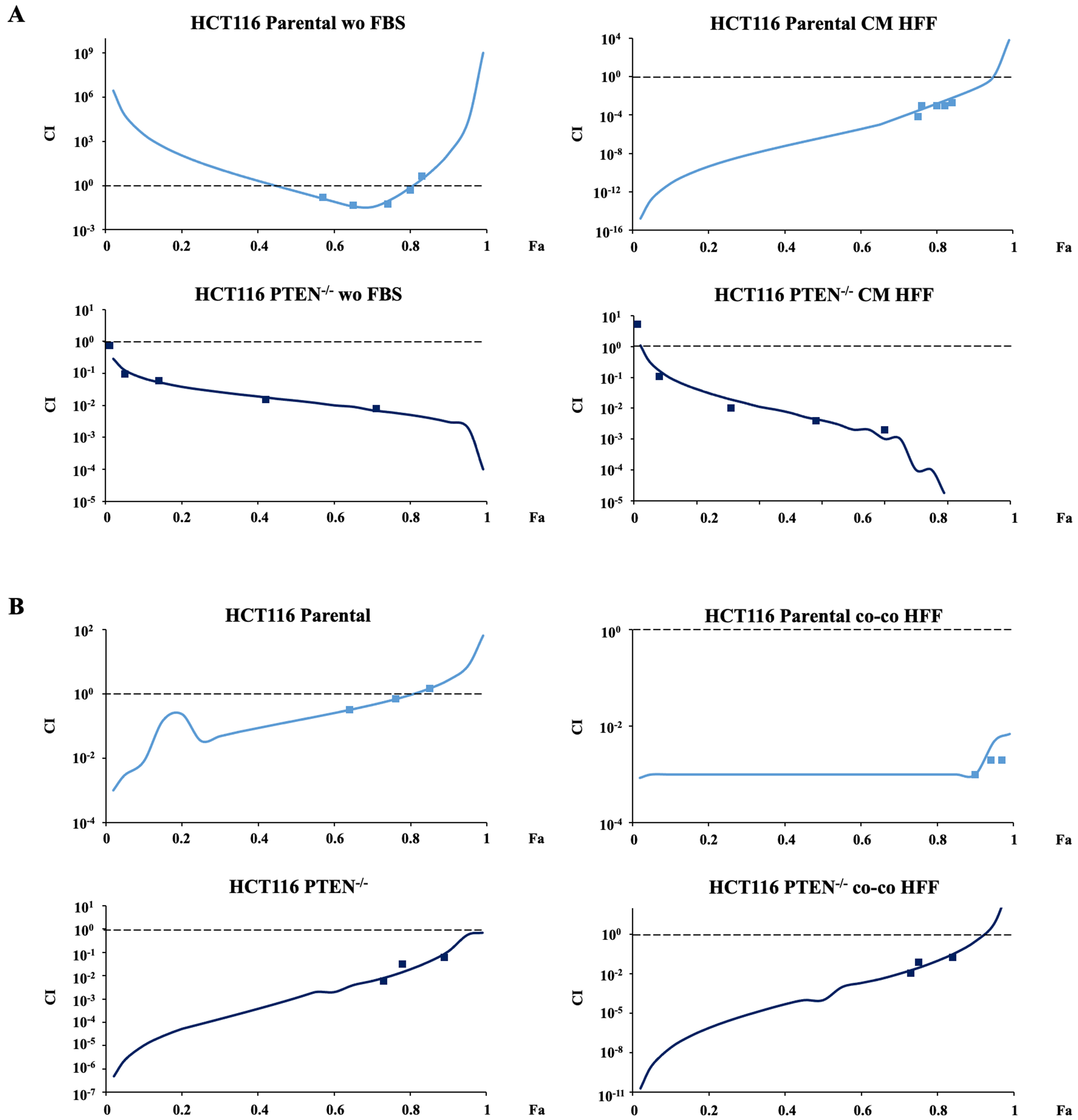

Supplementary Figure 2. Effect of combined Tram and Geda treatment in isogenic CRC cell lines in different condition of growth. While the response of HCT116 PTEN$^{-/-}$ cell line to combination treatment was independent of condition of growth, the response to combined treatment was strongly influenced by tumor microenvironment elements in PTEN-competent context. **A.** HCT116 Parental (top panels) and HCT116 PTEN$^{-/-}$ (bottom panels) cells were exposed to Tram and Geda in a fixed dose-ratio combination (1:1) in serum-free condition (left panels) or with fibroblast HFF-CM (right panels). **B.** HCT116 Parental (top panels) and HCT116 PTEN$^{-/-}$ (bottom panels) cells were exposed to Tram and Geda in a fixed dose-ratio combination (1:1) in wo FBS (left panels) or in co-culture with HFF (right panels). CI were calculated by

conservative isobologram analysis for experimental data and plotted against the fraction affected (Fa).

Supplementary Figure 3

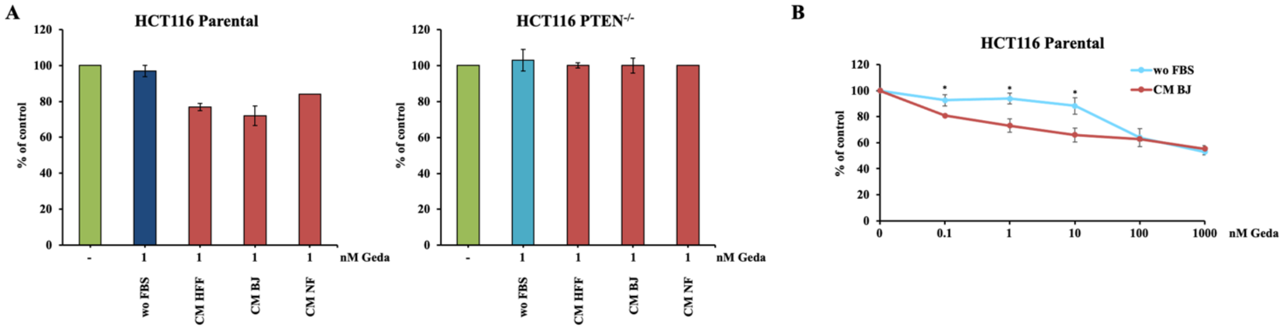

Supplementary Figure 3. Increased sensitivity to immortalized or normal colon fibroblast CM to Geda.

The response to double PI3K/mTOR inhibitor (Geda) was increased by different fibroblast-CM in PTEN-competent CRC cells.

A. HCT116 Parental and HCT116 PTEN$^{-/-}$ cell lines were treated with 1 nM of Geda in serum-free condition, fibroblast HFF-derived CM, fibroblast BJ-derived CM or normal fibroblast (NF)-derived CM. Cell viability were assessed by Crystal Violet assay after 72 h of treatment. Results are expressed as percentage of growth inhibition relative to untreated control and represent the average ± SEM of two independent experiments for serum free condition, HFF-CM and BJ-CM. Results of a representative experiment is shown for NF-CM. **B.** HCT116 Parental cell line was treated with increased doses (dose range 0.1-1000 nM) of Geda in serum-free condition or fibroblast BJ derived CM. Cell viability was assessed by Crystal Violet assay after 72 h of treatment. Results are expressed as percentage of growth inhibition relative to untreated control and represent the average ± SEM of three independent experiments. Asterisks indicate statistically significant differences (p-value < 0.05 by 2-tailed Student’s t test) for the comparison between treatment under wo FBS and BJ-CM conditions.

Supplementary Figure 4

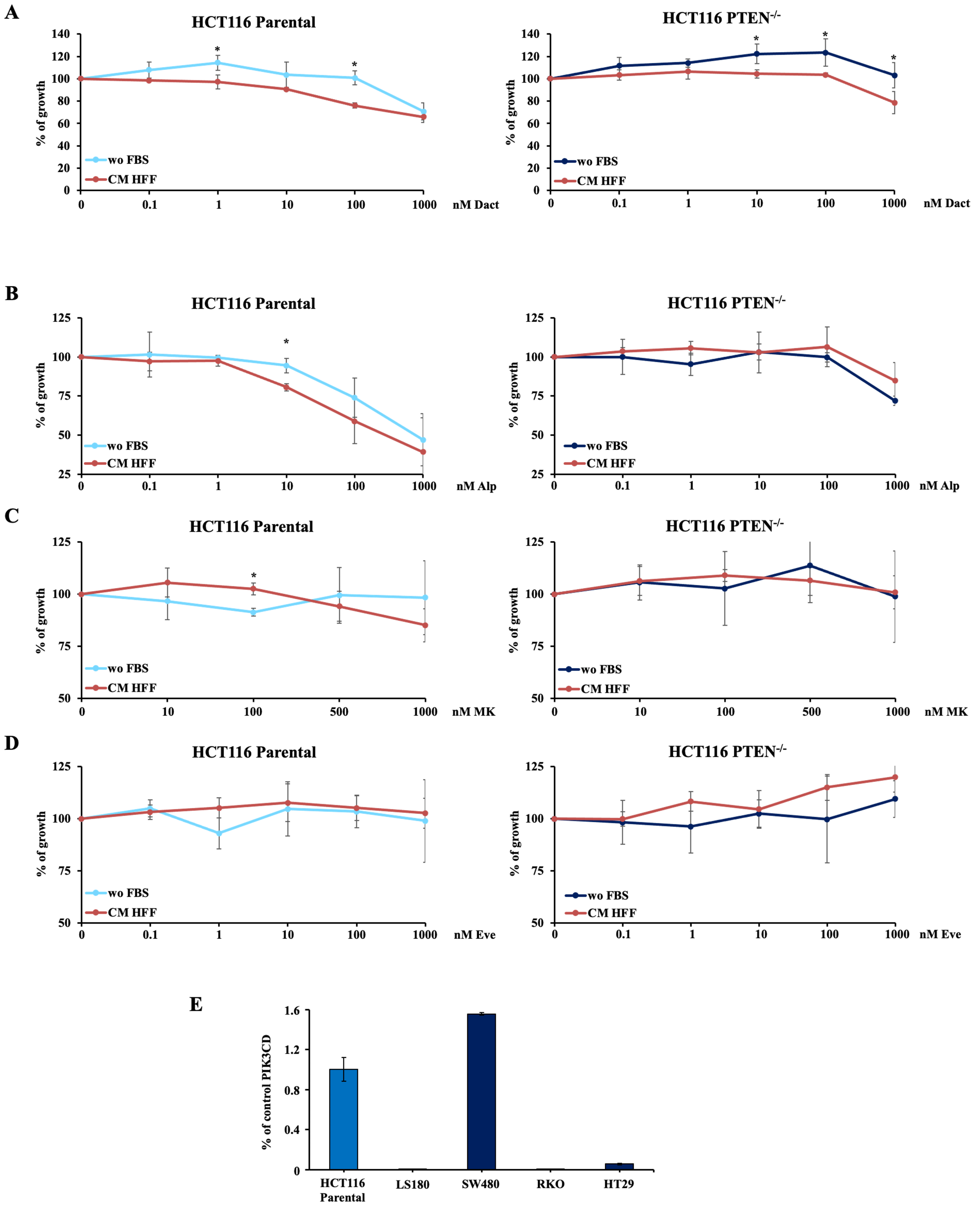

Supplementary Figure 4. Double PI3K/mTOR inhibition is necessary for the fibroblast-CM dependent increased sensitization in PTEN-competent CRC cell line. The sensitization to

Geda in the presence of fibroblast-CM was obtained only with double PI3K/mTOR inhibition. Indeed, no difference regardless of PTEN status or condition of growth was observed with single PI3K pathway inhibitors. HCT116 Parental and HCT116 PTEN$^{-/-}$ cell lines were treated with increased doses of Dact (dose range 0.1-1000 nM, **A**), PI3K inhibitor Alp (dose range 0.1-1000 nM, **B**), AKT inhibitor MK (dose range 10-1000 nM, **C**), mTOR inhibitor Eve (dose range 0.1-1000 nM, **D**) under serum-free condition or HFF-CM. Cell viability were assessed by Crystal Violet assay after 72 h of treatment. Results are expressed as percentage of growth inhibition relative to untreated control and represent the average ± SEM of three independent experiments. Asterisks indicate statistically significant differences (p-value < 0.05 by 2-tailed Student's t test) for the comparison between treatment under wo FBS and HFF-CM conditions.
E. The presence of PIK3CD was detected by RT-qPCR in HCT116 Parental, LS180, SW480, RKO and HT29 cell lines. Results were evaluated as ΔΔct of PIK3CD relative to RPL19 and expressed as the ratio assuming the levels in the control as 1.0. Results of a representative experiment performed is shown.

Supplementary Figure 5

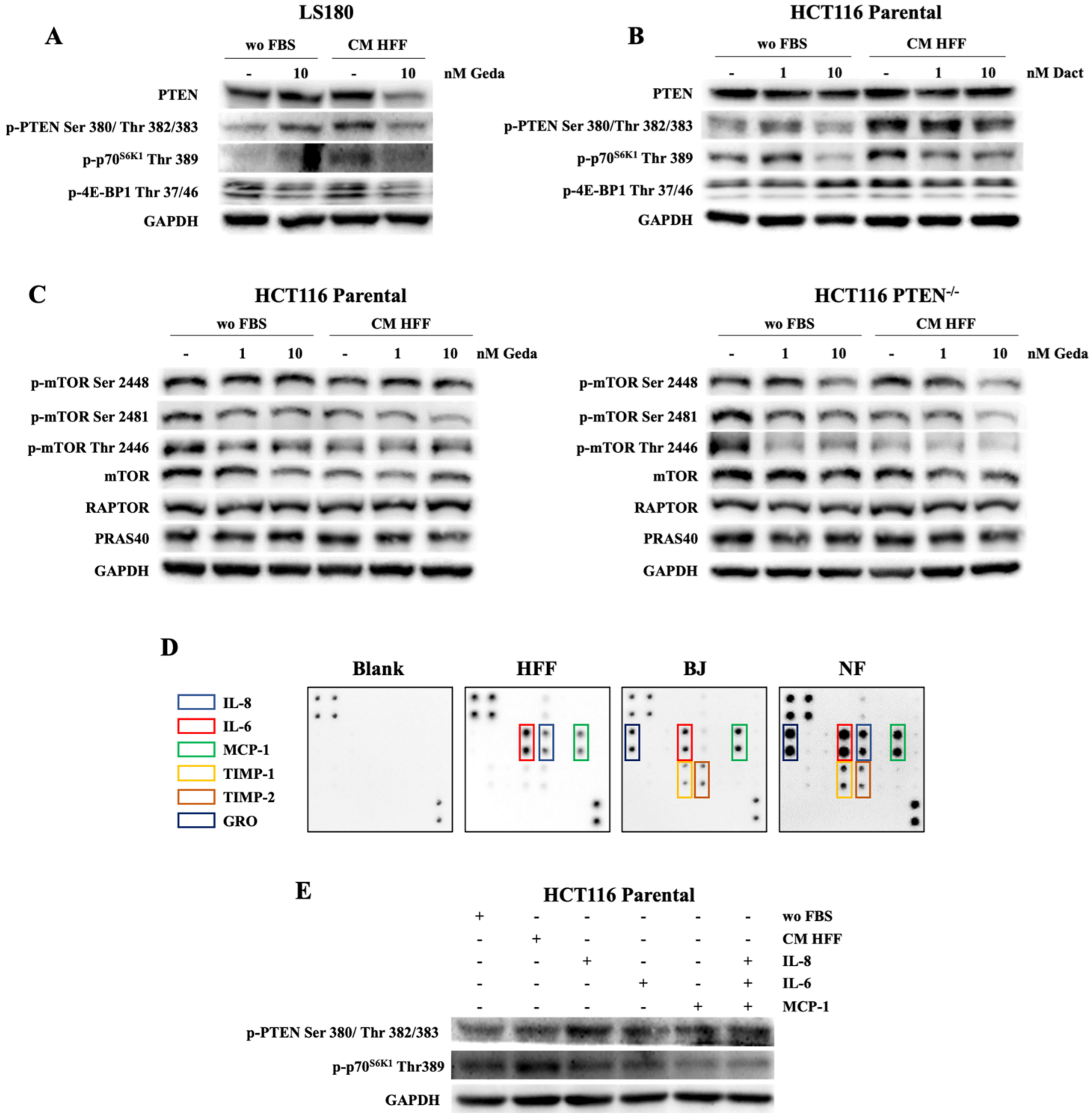

Supplementary Figure 5. PI3K signaling pathway activation after PI3K/mTOR inhibition and cytokine stimulation. The presence of fibroblast-CM induced a PI3K pathway activation thus leading to a higher sensitivity to Geda and Dact treatment in both HCT116 Parental and LS180 cell lines, as compared to serum-free condition **A**. LS180 cells were treated with 10 nM of Geda in serum-free condition and HFF CM. Cells were lysed and analyzed by Western Blotting using specific antibodies (as indicated). GAPDH is shown as protein loading and blotting control. **B.** HCT116 Parental cells were treated with 1 and 10 nM of Dact in serum-free condition and HFF CM. Cells were lysed and analyzed by Western Blotting using specific antibodies (as indicated). GAPDH is shown as protein loading and blotting control. **C.** HCT1116 Parental and HCT116 PTEN$^{-/-}$ cell lines were treated with 1 and 10 nM of Geda in serum-free condition and HFF CM. Cells were lysed and analyzed by Western

Blotting using specific antibodies (as indicated). GAPDH is shown as protein loading and blotting control.

Results of a representative experiment out of three independent experiments performed are shown.

Despite the presence of IL-8, IL-6 and MCP-1 in all the analyzed CM, these three soluble factors were not involved in PI3K pathway hyperactivation after CM stimulation. **D.** Cell culture media of HFF, BJ and NF were analyzed by Human Angiogenesis Antibody Array. **E.** HCT116 Parental cell line was exposed to serum-free condition, HFF-CM or wo FBS with recombinant IL-8, IL-6, MCP-1 or their combination, as indicated. Cells were lysed and analyzed by Western Blotting using specific antibodies (as indicated). GAPDH is shown as protein loading and blotting control. Results of a representative experiment out of three independent experiments performed are shown.

Supplementary Figure 6

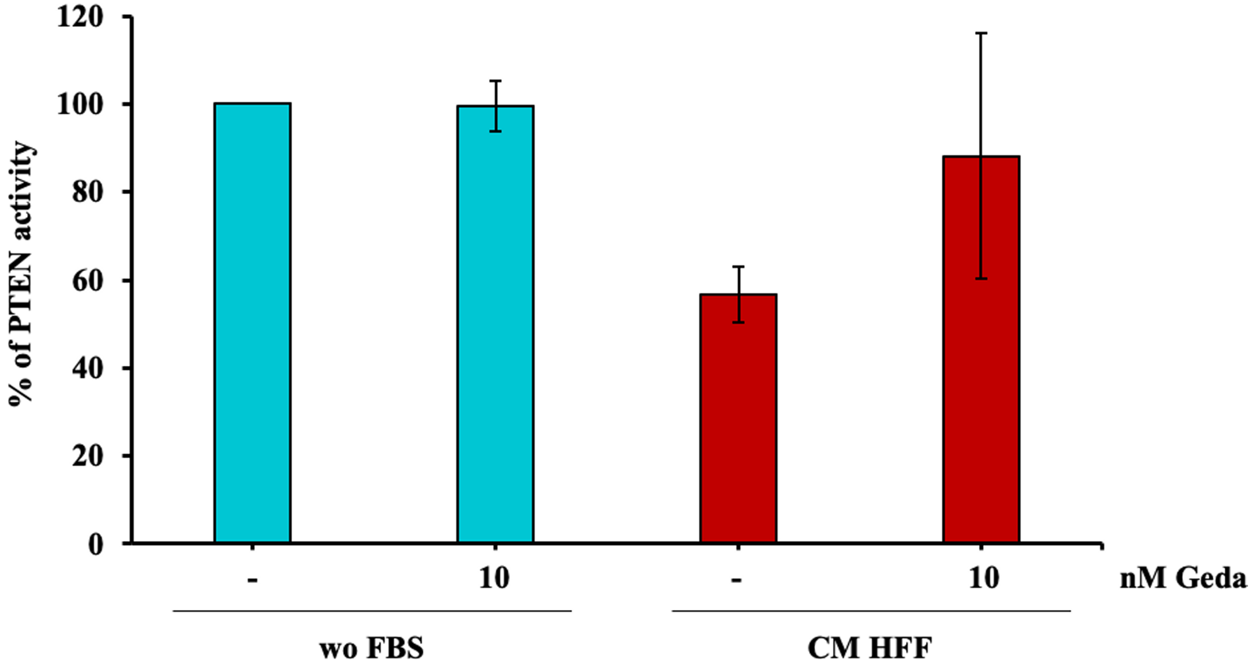

Supplementary Figure 6. Reduction of PTEN phosphatase activity in the presence of fibroblast-CM. HCT116 Parental cell line was treated with 10 nM of Geda in wo FBS and HFF-CM conditions. Results are expressed as percentage of PTEN activity relative to untreated wo FBS control. Results are expressed as percentage of growth inhibition relative to untreated control and represent the average ± SEM of three independent experiments.

3. *Supplementary Tables*

Supplementary Table 1. Ratio of half maximal inhibitory concentration (IC_{50}) of PI3K pathway inhibitors (nM) under CM HFF vs wo FBS condition of growth

Cell lines	Fold change of IC_{50} (nM)			
	**Dactolisib**	**Alpelisib**	**MK-2206**	**Everolimus**
**HCT116 Parental**	0.21	>1	0.21*	1
**HCT116 PTEN$^{-/-}$**	>1	>1	1	1

*IC_{50} value > 10^5 for both the conditions of growth.

4. *Original Images for Western Blot*

Figure 3A

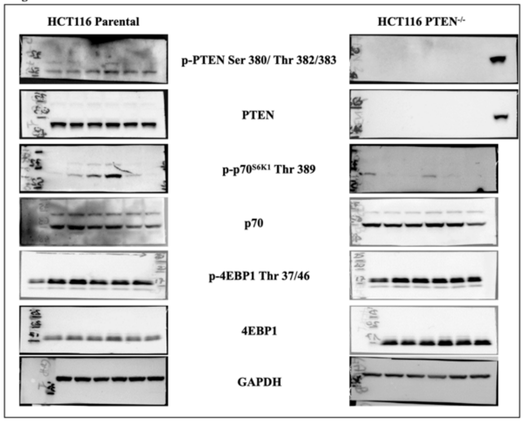

Figure 3B

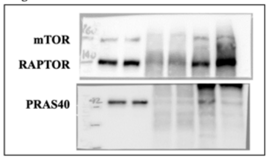

Figure 4B

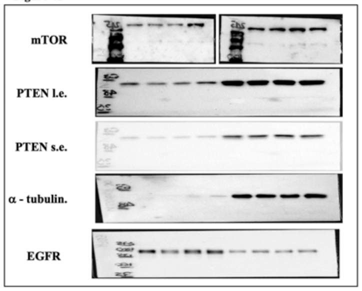

Figure 5A

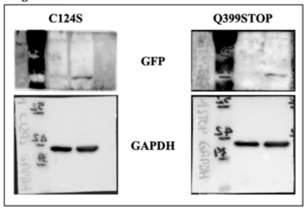

Figure 5B

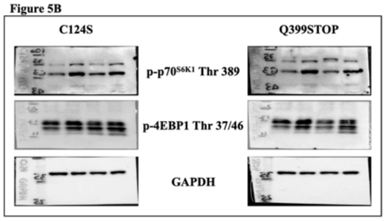

Supplementary Figure 5A

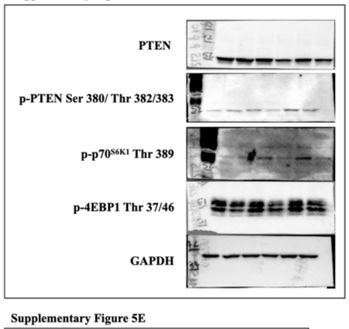

Supplementary Figure 5B

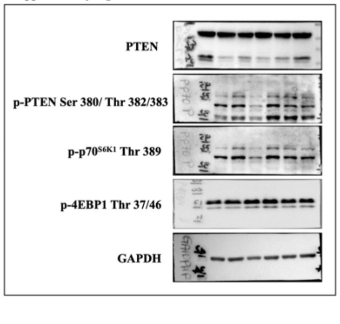

Supplementary Figure 5C

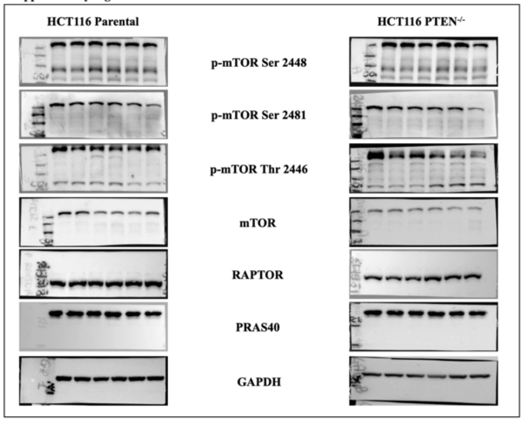

Supplementary Figure 5E

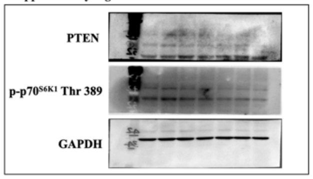

Supplement: Supplementary file 1 [file DataSheet_1.pdf]
